# Is the health of people living in rural areas different from those in cities? Evidence from routine data linked with the Scottish Health Survey

**DOI:** 10.1186/1472-6963-12-43

**Published:** 2012-02-17

**Authors:** P Teckle, P Hannaford, M Sutton

**Affiliations:** 1 Canadian Centre for Applied Research in Cancer Control (ARCC) "Advancing health economics, services, policy and ethics", #2-111, 675 West 10th Avenue, Cancer Research Centre, V5Z 1L3, Vancouver, BC, Canada; 2 University of Aberdeen, Aberdeen, UK; 3 University of Manchester, Manchester, UK; 4 Faculty of Medicine, School of Population and Public Health, University of British Columbia, Vancouver, BC, Canada

**Keywords:** Health determinants, Rural health, Administrative data-linkage, Survey methods

## Abstract

**Background:**

To examine the association between rurality and health in Scotland, after adjusting for differences in individual and practice characteristics.

**Methods:**

Design: Mortality and hospital record data linked to two cross sectional health surveys. Setting: Respondents in the community-based 1995 and 1998 Scottish Health Survey who consented to record-linkage follow-up. Main outcome measures: Hypertension, all-cause premature mortality, total hospital stays and admissions due to coronary heart disease (CHD).

**Results:**

Older age and lower social class were strongly associated with an increased risk of each of the four health outcomes measured. After adjustment for individual and practice characteristics, no consistent pattern of better or poorer health in people living in rural areas was found, compared to primary cities. However, individuals living in remote small towns had a lower risk of a hospital admission for CHD and those in very remote rural had lower mortality, both compared with those living in primary cities.

**Conclusion:**

This study has shown how linked data can be used to explore the possible influence of area of residence on health. We were unable to find a consistent pattern that people living in rural areas have materially different health to that of those living in primary cities. Instead, we found stronger relationships between compositional determinants (age, gender and socio-economic status) and health than contextual factors (including rurality).

## Background

Rural Scotland comprises 89% of Scotland's landmass, and contains 20% of the population, and 27% of those employed [1]. There is growing interest in the health of people living in rural and remote areas, and in the study of health care services provided to them [2-4]. Urban-rural variations in health outcomes have been studied within Scotland [5-9] and the UK [10,11]. Studies so far have used a variety of health outcomes, including long standing illness [12], mortality [13], cancer [14-16], hypertension and cardiovascular disease [17-20], and respiratory health [5]. The available evidence is derived from specific, one-off projects and the collective evidence is inconclusive [5,16,21-23]. Some studies in the US, Australia, and Canada have found rural residents to be in poorer health than their urban counterparts [24-26]. In Scotland, there is little evidence of important widespread urban-rural differences. A recent literature review about urban-rural health status differentials in developed countries suggested that rurality per se is not associated with poor health, but rural location is a major determinant of the nature, level of access to, and provision of health services [27].

The Scottish NHS resources allocation formula (The Arbuthnott Allocation Formula) is the first in the UK to include a cost adjustment for remoteness and rurality [28]. And the Kerr report highlighted that the rural population tends to have a significant proportion of older people who often have chronic diseases and do require more health care [29]. There is however, limited empirical evidence to indicate whether health outcomes are significantly different between rural and urban Scotland. Using objective health outcome measures, this study will shed light if there are significant differences between people living in rural and urban areas in Scotland.

Most of the previous studies were small area variation analyses and did not include the structure of the healthcare (especially primary care) serving them. Thus, they did not control for either the characteristics of individuals and/or the healthcare services received, either of which might influence health outcomes. Previous studies have suggested that geographical and organizational variations in the structure of primary care services should be considered in studies of health outcomes and health care services [30]. General practices vary in their structural characteristics, their use of health service resources, their standards of clinical care, and in patient outcomes. Structural characteristics of general practices, such as: the partnership size [31-33]; whether the practitioner works single-handedly; the age and sex of the general practitioner [34-36]; whether the practice provides vocational training [37] or engages in undergraduate medical education [38]; the size and characteristics of the practice list [39-41]; and whether the practice is in a rural location [42,43], have been found to be associated either with process of health care, or with different health outcomes or health care utilization [44-48]. There are few female GPs working in rural areas and this leads to reduced choice for patients and poorer access to some treatments [49-51].

The characteristics of the population served as well as the practices providing care are complex confounders that previous studies have not adequately accounted for. Information from such studies are of limited use for health care planners, who require representative, regularly updated information, ideally collected for a number of purposes in order to reduce administration costs.

The rationale for this study therefore was to assess whether routinely available national datasets can be used to examine whether there are rural-urban variations in health outcomes, after allowing for differences in the characteristics of the populations, the practices serving them, and the place of residence of the population.

To control for the characteristics of the population and the practices, we need data on the socio-demographic characteristics of the population, characteristics of the general practices serving them, and data on the rural-urban classification of the residence of the population. It is not easy to get such data due to logistical and ethical issues. That is why previous studies on this issue linked different databases to create a dataset for analysis. Most of these studies, however, used aggregated area-based indicators of population health and structure of care [52]. Such aggregation might mask the characteristics of individual general practitioners or practices and thereby affect the results. In epidemiological term, such approaches are prone to ecological fallacy, i.e. area-level aggregate results do not necessary mean that relationships hold at the individual level. Individual level data avoids the problem of ecological fallacy which can arise when area-based data are used [53].

In 2004, a record linkage exercise was undertaken by the Information Services Division (ISD) of NHS Scotland to link both the 1995 and 1998 Scottish Health Survey data to the linked Scottish hospital admission and mortality database (SMR). Its creation provided an ideal opportunity to examine the association between rurality and health outcomes. In order to adjust for some aspects of the structure of the primary care, we have linked this data to the Scottish General Practitioners Census Data (SGPC).

The main focus of the Scottish Health Survey (SHS) was on cardiovascular disease. We therefore included admissions due to coronary heart disease (CHD), total hospital stays and hypertension as health outcome measures. The SHS is linked to death records from the General Register Office for Scotland. We therefore included mortality which is usually used as an overall health outcome indicator.

## Methods

### Data sources

#### Individual characteristics

The Scottish Health Survey (SHS) studied a nationally representative sample of people living in private households in Scotland; (n = 7,932 adults aged 16-64 in 1995; and n = 9,047 adults aged 16-74 and children aged 2-15 in 1998) [54]. In both years a particular focus was on cardiovascular disease (CVD) and related risk factors. Each survey collected information about the demographic and socio-economic characteristics of participants, including: age, gender, social class, education and housing tenure. Both surveys are claimed to be the first reliable surveys in Scotland that give a comprehensive picture of the health of the whole population, its biological characteristics and health-related behavior [55]. The surveys were made available through the UK Data Archive at the University of Essex.

The overall aim of the surveys was to estimate the prevalence of certain health conditions and risk factors in Scotland, and to monitor progress towards health and dietary targets [54]. The surveys were conducted by the Joint Health Surveys Unit of the National Centre for Social Research (formerly Social Community Planning Research), and the Department of Epidemiology and Public Health at University College London (UCL). To enable regional comparisons the survey divided Scotland into seven regions by aggregating health boards. Both surveys employed stratified, multi-stage random sampling to provide a nationally representative sample[55].

Of those interviewed in 1995, 6,958 informants (and 7,455 adults and 3,211 children in 1998) were subsequently visited by a nurse. Within these visits, at least one usable blood sample was taken from 6,184 adults (in 1995) and from 6,178 adults and 466 children aged 11-15 in 1998 informants. The Information Services Division (ISD) of National Health Services (NHS) Scotland linked the Scottish Health Survey to the Scottish Morbidity Records and made the data available for this study. These data were used to answer our research question in this study.

#### Definition of rurality

There is no universally agreed definition of what constitutes an 'urban' or 'rural' area [56]. Rurality of where individuals lived were measured using the Scottish Executive Urban Rural Classification (SEURC) [57].

The SEURC was selected as a pragmatic definition of rurality in this study. Its advantages include: it takes on board several indicators that are likely to be associated with economic issues, such as the dispersed nature of the population lacking economies of scale and large travelling times affecting access to care; it is available at national level enabling linkage to other routinely collected national datasets base to examine the relationship between rurality, health, health care provision and utilization; it is an appropriate generic definition to describe the variance in the characteristics of the populations living in remote rural areas, the practices serving them, and variations in health and health care between rural and urban areas; it enables analyzing individuals in terms of their rurality and urbanity versus their remoteness and accessibility [57]. The SEURC is being increasingly used in many studies [6,8,9,22,58,59]

The SEURC classification divides Scotland into eight categories based on settlement size and remoteness: *Primary Cities *(settlements with over 125,000 people); *Urban Settlements *(settlements with between 10,000 and 124,999 people); *Accessible Small Towns *(settlements with between 3,000 and 10,000 people and within 30 minute drive time of a settlement of 10,000 or more); *Remote Small Towns *(settlements with between 3,000 and 10,000 people and between 30 and 60 minutes drive time of a settlement of 10,000 or more); *Very Remote Small Towns *(settlements with between 3,000 and 10,000 people and more than 60 minute drive time away from a settlement of 10,000 or more); *Accessible Rural *(settlements of less than 3,000 people and within a 30 minute drive time of a settlement of 10,000 or more); *Remote Rural *(settlements of less than 3,000 people and between 30 and 60 minutes drive time of a settlement of 10,000 or more) and *Very Remote Rural *(settlements of less than 3,000 people and more than 60 minute drive time of a settlement of 10,000 or more).

#### Health outcomes

As part of the survey, a nurse took three blood pressure measures for all individuals who gave consent. We used the mean of the three measures and defined people as hypertensive if their systolic blood pressure was ≥ 150 mmHg, or diastolic blood pressure was ≥ 90 mmHg. This definition was taken from the New General Medical Services contract - Quality of Outcome Framework criteria for hypertension [60]. Death records from the General Register Office for Scotland were used to identify all those in the sample who had died by November 2006. Scottish Morbidity Recording (SMR) data routinely collected by ISD, concerning general acute hospital inpatient and day case episodes provided information about hospital admissions due to coronary heart disease (CHD) and total number of days spent in hospital up to November 2006. SMR data go back to January 1981. We used weights provided by the surveys to account for the sampling design and non-response bias.

#### Practice characteristics

The ISD maintains a dataset of all doctors working as general practitioners in Scotland. For the years 1996 and 1998, we obtained the age and sex of each general practitioner (GP), the size of the partnership that each doctor worked in, the contracted time commitment of each principal (expressed as whole time equivalent -WTE), and the number, age, and gender of people registered with each practitioner. We have linked the Scottish General Practitioners Census to the survey using the serial number and practice code pertaining for each participant in the SHS at the time of the survey. (See details in "Linkage" section) We also ascertained whether the practice had a GP vacancy. We calculated practice-level partnership size and availability of a female practitioner.

### Linkage

Everyone participating in the Health Surveys was asked to give explicit consent to their information being passed to ISD for subsequent linkage to routinely available datasets. For those who gave such permission, their health survey information was linked by ISD, on our behalf, to the SMR data. This was done using standard probability matching, based on name, postcode and date of birth. The postcode of each individual was also used by ISD to assign the appropriate SEURC category. The subsequent Scottish Health Survey-linked-to Scottish Morbidity Data (SHS-SMR) dataset was stripped of any identifying information before being released to us. ISD also provided the serial number and practice code pertaining for each participant in the SHS at the time of the survey. This enabled us to link the new SHS-SMR information to the characteristics of each participant's practice.

Our analysis was limited to 15668 survey respondents who said that their information could be linked to the administrative datasets (Table 1). The ISD managed to link 7363 adults aged 16-64 (1995 survey) and 8305 adults aged 16-74 (1998 survey). The linked dataset contained mortality data from 1995 up to November 2006 and hospital admission data for CHD from 1981 up to November 2006.

**Table 1:** Percent distributions and mean (sd) for individual and practice variables by SEURC

SEURC1	PC%	US%	AST%	RST%	RRST%	AR%	RR%	VRR%	TOTAL
N	5427	4900	1757	434	296	1649	548	645	15668
Age (sd)	42.06 (14.9)	42.62 (14.9)	43.35 (15.1)	45.01 (15.5)	43.25 (15.1)	43.95 (14.5)	44.48 (14.9)	45.11 (14.7)	42.90 (14.9)
Female	56.4	55.9	54.1	59.0	53.7	55.1	49.3	54.3	0.56
**Social class**									
professional	5.3	3.1	2.6	3.5	3.3	4.2	4.8	2.1	4.0
intermediate	24.6	20.9	23.6	21.8	18.4	30.9	30.5	30.0	24.3
Skilled non-manual	24.2	23.8	22.2	21.3	23.9	19.9	16.8	21.7	23.0
Skilled manual	19.9	22.8	23.3	21.6	25.7	21.8	21.6	22.3	21.7
Partly-skilled	16.7	20.3	18.5	19.9	17.6	15.8	19.1	14.6	18.0
unskilled	8.3	8.2	8.3	10.4	11.0	6.5	6.6	8.5	8.1
**House tenure**									
own	57.4	63.2	63.6	59.4	59.8	66.2	65.8	70.2	61.7
public	34.7	33.0	32.5	35.5	33.8	18.8	14.3	18.6	30.9
private	6.8	2.9	3.1	4.1	4.1	9.2	11.2	6.5	5.4
others	1.2	0.9	0.8	0.9	2.4	5.9	8.8	4.7	2.0
**Health outcomes**									
Hypertensive	14.4	14.7	16.0	13.0	20.0	16.5	15.5	16.5	15.1
All-cause death	6.4	6.1	5.8	7.1	5.7	5.9	4.2	4.2	6.0
CHD admissions	4.0	4.5	4.0	3.0	4.0	4.0	4.0	4.4	4.1
Hospital stays2	47.0	50.0	47.0	46.0	55.0	44.0	47.0	51.0	48.2
**Practice char**									
wtegps1	4.74	11.2	8.6	8.7 (6.4)	5.55	7.78	9.6	9.4	8.01
000_3 _(sd)	(19.4)	(73.5)	(40.0)		(3.6)	(41.0)	(17.5)	(22.5)	(47.1)
Gpage	42.66	42.84	43.12	41.36	41.06	42.52	42.13	43.09	42.68
(sd)	(4.7)	(4.3)	(4.4)	(4.1)	(4.5)	(4.4)	(3.7)	(5.8)	(4.54)

^1 ^*SEURC *Scottish Executive Urban Rural Classification, *PC *Primary Cities, *US *Urban Settlements, *AST *Accessible Small Towns, *RST *Remote Small Towns, *VRST *Very Remote Small Towns, *AR *Accessible Rural, *RR *Remote Rural, *VRR *Very Remote Rural. ^2 ^At least one day or more hospital stays 3, *WTE GPs per 1000 *Whole Time Equivalent GPs per 1000 population

We were unable to assign a practice to 146 (1.9%) respondents in the 1995 SHS and 87 (1.1%) respondents in the 1998 survey. These individuals were excluded from the analyses. Our analysis was restricted to respondents who were known to be registered with a practice at the time of survey. This enabled us to examine any association between practice characteristics and health. From the available hospitalization data, only the time of the first event is known. We ran separate models, one for all respondents and one excluding respondents who had an event prior to the survey. Comparison of the two models showed no significant difference in the pattern observed, so we have shown results using all available information for respondents.

### Analysis

We used hierarchical models to account for the stratified, multi-stage random sampling used in both surveys to provide a nationally representative sample [55]. Observations are nested within seven regions and within 983 general practices. We employed a hierarchical model to account for clustering.

In analyzing the data, we used sampling weights from the survey to account for the sampling design (non-probabilistic) and non-response bias. This provided robust standard errors that relax the assumption of homesckedaciticty and adjust for heterosckedacitcity [61]. The surveys over-sampled rural areas in order to provide sufficient sample sizes within each region.

For each health outcome, we started with a basic model which adjusted for age and gender only. We created five-year age bands to capture the specific effect of age in men and women. Males < 30 years was taken as the reference group. Place of residence was then introduced to examine age-sex standardized differences in outcome between people living in different parts of Scotland. We then introduced socio-economic characteristics. We included a year dummy to capture change in health outcome measures over time, with year 1995 as the base year. The final model also adjusted for practice characteristics including WTE of GPs per 1000 population, and mean GP age. Additional variables about the structure of the practices were then included to see if the model was improved; availability of at least one female GP, whether the practice had a GP vacancy, and practice-level partnership size (total number of GPs in a practice). We compared the models using the Akaike information criterion (AIC) and Bayesian information criterion (BIC), and using 'fitstat' command in Stata.

We used the log likelihood, AIC and BIC to compare the performance of these models. The AIC is defined as *AIC *= -2*ln L*+2*k*, and the BIC is defined as *BIC *= -2*ln L*+*k ln(N)*, where *ln L *is the maximized log likelihood of the model and *k *is the number of parameters in the mode, and *N *is the sample size. We preferred the model with larger values of the log likelihood and smaller values of AIC and BIC. We have not presented these results due to space limitation but they are available from the authors. All three criteria favoured the model with WTE of GPs per 1000 population and mean age of GPs in a practice. Including practice structural variables (availability of a female GP, GP vacancy, and number of GPs in a practice) did not improve the model. This could probably be due to WTE of GPs per 1000 population, and mean GP age capturing the significant characteristics of a practice.

The number of observations was reduced when the survey data was linked to the practice and hospitalization (SMR) data. To reduce the probability of getting statistically significant associations by chance (type I error), a small significance level (*p *< 0.01) was used [62]. The study did not require ethical approval as respondents were not identifiable.

A set of eight urban-rural category dummy variables were created for rurality. Summary statistics for urban-rural categories were compared using the *t*-test, with Primary Cities (the largest group) as the reference group. The joint significance of differences between categories was measured using the F-test, with seven degrees of freedom.

The dependent variables, prevalence of hypertension and all-cause mortality, were dichotomous variables, while hospital admissions due to CHD and total number of hospital admissions were non-negative count values. For the dichotomous variables, multivariate logit models were generated.

For the non-negative integer count variables, negative binomial regression was used. The analyses used Stata version 11.0 [63].

## Results

The distribution of the population across the eight geographic categories and socio-demographic characteristics by survey year is presented in Table 1. The three rural categories contained 12% of the total survey respondents. The pooled dataset consisted of 15,668 subjects (7363 participants in the 1995 and 8305 in 1998 survey: Table 1). The average age (standard deviation: sd) was 42.9 (14.9) years. Both surveys had a similar distribution of participants with respect to gender, social class, housing tenure and place of residence. The characteristics of the practices serving the survey participants were also similar in the two surveys: GP principals working in rural areas tended to be older, male and single-handed, when compared with those in primary cities (data not shown).

The mean (sd) systolic and diastolic blood pressure of participants was 129.8 (18.0) mmHg and 71.7 (12.0) mmHg respectively. Overall, 2027 (15%) of participants were classified as hypertensive, 942 (6%) died, 642(4%) were admitted to hospital for CHD and 7551 (48.2%) had spent at least one day or more in hospital.

### Hypertension

Older people were more likely to be hypertensive in both sexes (Table 2 Model 1). Adjusting for rurality, individuals living in remote small towns were less likely to have hypertension than those living in primary cities (OR = 0.57, 99% CI 0.33 to 1.00, *p *= 0.01: Table 2 Model 2). This relationship did not persist when we control for the socio-economic differences. Living in publicly owned housing was highly associated with an increased probability of hypertension (OR = 1.34, 99% CI 1.11 to 1.60, *p *< 0.001: Table 2 Model 3). When practice characteristics and year dummies were introduced, the significant association between living in remote small towns and hypertension did not reach our threshold for statistical significance (*p *= 0.096). Being older and living in publicly owned housing was associated with a statistically significant higher likelihood of hypertension in the fully adjusted model.

**Table 2:** Association between hypertension and location, without and with adjustments for population and practice characteristics

Hypertension		Model 1			Model 2			Model 3			Model 4 2	
	OR*	**(99% CI)**	P > z	OR	**(99% CI)**	P > z	OR	**(99% CI)**	P > z	OR	**(99% CI)**	P > z
**Age and sex**												
Male < 30	ref.			ref.			ref.			ref.		
Male30_34	1.86	(0.96 3.58)	0.015	1.88	(0.97 3.62)	0.013	1.77	(0.88 3.54)	0.036	1.90	(0.93 3.86)	0.020
Male35_39	2.52	(1.34 4.75)	< 0.001	2.52	(1.34 4.75)	< 0.001	2.34	(1.19 4.59)	0.001	2.30	(1.15 4.62)	0.002
Male40_44	5.29	(2.98 9.41)	< 0.001	5.30	(2.98 9.44)	< 0.001	5.20	(2.76 9.80)	< 0.001	5.14	(2.68 9.86)	< 0.001
Male45_49	7.16	(4.03 12.71)	< 0.001	7.21	(4.06 12.79)	< 0.001	6.72	(3.58 12.61)	< 0.001	7.33	(3.84 13.96)	< 0.001
Male50_54	11.08	(6.43 19.07)	< 0.001	11.09	(6.44 19.12)	< 0.001	10.71	(5.89 19.49)	< 0.001	11.24	(6.09 20.75)	< 0.001
Male55_59	13.91	(8.02 24.11)	< 0.001	14.01	(8.08 24.30)	< 0.001	13.41	(7.39 24.33)	< 0.001	13.51	(7.34 24.87)	< 0.001
Male60_64	18.55	(10.86 31.70)	< 0.001	18.67	(10.92 31.94)	< 0.001	17.53	(9.81 31.35)	< 0.001	19.35	(10.67 35.11)	< 0.001
Male65_69	22.00	(12.20 39.66)	< 0.001	22.46	(12.45 40.51)	< 0.001	20.91	(11.07 39.47)	< 0.001	23.11	(11.86 45.04)	< 0.001
Male70_74	22.45	(11.88 42.42)	< 0.001	22.89	(12.11 43.25)	< 0.001	21.01	(10.63 41.50)	< 0.001	20.89	(10.36 42.12)	< 0.001
Female < 30	0.33	(0.12 0.90)	0.004	0.33	(0.12 0.90)	0.005	0.28	(0.09 0.89)	0.005	0.33	(0.10 1.07)	0.015
Female30_34	0.45	(0.17 1.16)	0.030	0.45	(0.18 1.17)	0.031	0.38	(0.14 1.06)	0.016	0.40	(0.14 1.15)	0.025
Female35_39	1.15	(0.58 2.29)	0.589	1.16	(0.59 2.31)	0.571	1.13	(0.53 2.39)	0.675	1.14	(0.52 2.50)	0.657
Female40_44	1.90	(1.00 3.60)	0.010	1.91	(1.01 3.62)	0.009	1.81	(0.90 3.61)	0.028	1.84	(0.86 3.92)	0.039
Female45_49	3.75	(2.09 6.72)	< 0.001	3.78	(2.10 6.79)	< 0.001	3.57	(1.89 6.77)	< 0.001	3.56	(1.86 6.82)	< 0.001
Female50_54	7.14	(4.06 12.55)	< 0.001	7.18	(4.09 12.62)	< 0.001	6.60	(3.52 12.38)	< 0.001	7.19	(3.83 13.51)	< 0.001
Female55_59	9.37	(5.37 16.34)	< 0.001	9.49	(5.44 16.55)	< 0.001	8.75	(4.71 16.25)	< 0.001	9.09	(4.83 17.09)	< 0.001
Female60_64	16.30	(9.50 27.97)	< 0.001	16.31	(9.48 28.07)	< 0.001	14.19	(7.65 26.29)	< 0.001	14.88	(7.90 28.01)	< 0.001
Female65_69	21.90	(12.35 38.82)	< 0.001	22.20	(12.50 39.44)	< 0.001	19.77	(10.42 37.49)	< 0.001	20.90	(10.69 40.88)	< 0.001
Female70_74	35.42	(19.90 63.05)	< 0.001	36.02	(20.22 64.18)	< 0.001	32.74	(17.26 62.13)	< 0.001	33.11	(17.35 63.17)	< 0.001
**Location**1												
PC				ref.			ref.			ref.		
US				1.01	(0.84 1.21)	0.925	1.04	(0.86 1.26)	0.573	1.06	(0.87 1.29)	0.452
AST				1.03	(0.78 1.37)	0.758	1.06	(0.80 1.40)	0.602	1.09	(0.82 1.45)	0.458
RST				0.57	(0.33 1.00)	0.010	0.60	(0.35 1.05)	0.018	0.68	(0.37 1.24)	0.096
VRST				1.40	(0.86 2.27)	0.074	1.59	(1.03 2.45)	0.006	1.50	(0.95 2.35)	0.021
AR				0.96	(0.74 1.25)	0.682	1.02	(0.78 1.34)	0.868	1.03	(0.77 1.37)	0.809
RR				0.99	(0.63 1.56)	0.949	1.04	(0.65 1.64)	0.845	1.08	(0.68 1.72)	0.655
VRR				1.05	(0.76 1.46)	0.679	1.17	(0.84 1.63)	0.234	1.09	(0.75 1.60)	0.542
**Social Class**												
Intermediate				ref.	ref.							
Professional							1.18	(0.78 1.80)	0.305	1.12	(0.72 1.73)	0.506
Skilled non-manual							1.14	(0.89 1.46)	0.110	1.12	(0.86 1.45)	0.279
Skilled manual							1.13	(0.89 1.43)	0.146	1.17	(0.91 1.51)	0.100
Partly skilled							1.10	(0.86 1.40)	0.335	1.14	(0.88 1.48)	0.190
Unskilled							1.10	(0.81 1.50)	0.409	1.13	(0.81 1.57)	0.338
**House ownership**												
Owned							ref.			ref.		
Publicly rented							1.34	(1.11 1.60)	< 0.001	1.35	(1.11 1.63)	< 0.001
Privately rented							1.08	(0.66 1.60)	0.690	1.13	(0.68 1.87)	0.528
Others							1.09	(0.57 2.10)	0.721	1.15	(0.57 2.29)	0.607
**Practice char**.												
WTE GPs per 1000 pop.										0.83	(0.49 1.40)	0.361
GPs age (mean)										0.99	(0.98 1.01)	0.340
Survey year 1998										0.98	(0.81 1.19)	0.822
Log pseudo-likelihood	-4135			-4127			-3931			-3541		
Sample size	13227			13216			12424			11193		
Joint significance of rural										X^2^(7) = 8.19	p = 0.31 6	

* *OR *Odds Ratio *ref*. reference group ^1 ^*PC *Primary Cities, *US *Urban Settlements, *AST *Accessible Small Towns, *RST *Remote Small Towns, *VRST *Very Remote Small Towns, *AR *Accessible Rural, *RR *Remote Rural, *VRR *Very Remote Rural. ^2 ^*p*-values calculated using standard errors clustered by practice

None of the practice characteristics were associated with hypertension (Table 2 Model 4). The joint significance test indicated no significant variation in the chances of hypertension across the eight urban rural categories.

### Mortality

In the first model, older males and females were more likely to die males less than 30 years old (Table 3 Model 1). The risk estimates for those aged fifty years and older were highly significant (*p *< 0.001). After adjustment for rurality, older age remained strongly associated with mortality. Individuals in remote rural (OR = 0.51, 99% CI 0.30 to 0.87, *p *< 0.01) and very remote rural (OR = 0.45, 99% CI 0.25 to 0.81, *p *< 0.001) were less likely to die than those in primary cities (Model 2). After allowing for the social status and housing tenure of individuals, those in very remote rural areas still had a lower chance of dying, but the strength of the association was diminished slightly. Individuals with the highest socio-economic status (professionals) had significantly lower likelihood of mortality than the intermediate socio-economic group (Table 3 Model 3). Living in publicly owned housing was associated with higher mortality. The final model that adjusted for the practice characteristics indicated that individuals in very remote rural areas have lower mortality compared to primary cities. Older age and lower social class remained significantly associated with mortality. Indeed the relationships with social class increased (in terms of magnitude and significance) after adjustment for practice characteristics.

**Table 3:** Association between all-cause mortality and location, without and with adjustments for population and practice characteristics

	Model 1			Model 2			Model 3			Model 4		
	OR*	(99% CI)	P > z	OR	(99% CI)	P > z	OR	(99% CI)	P > z	OR	(99% CI)	P > z
**Age and sex**												
Male < 30	ref.			ref.			ref.			ref.		
Male30_34	1.62	(0.43 6.12)	0.352	1.64	(0.43 6.20)	0.340	2.27	(0.47 11.02)	0.181	1.80	(0.34 9.54)	0.363
Male35_39	1.62	(0.46 5.67)	0.323	1.62	(0.46 5.66)	0.324	2.40	(0.55 10.49)	0.125	2.43	(0.56 10.63)	0.120
Male40_44	3.13	(1.00 9.81)	0.010	3.16	(1.00 9.93)	0.010	4.72	(1.13 19.74)	0.005	4.24	(0.99 18.14)	0.010
Male45_49	4.52	(1.50 13.57)	< 0.001	4.57	(1.52 13.74)	< 0.001	6.63	(1.65 26.61)	< 0.001	5.82	(1.42 23.78)	0.001
Male50_54	12.25	(4.41 33.99)	< 0.001	12.36	(4.44 34.37)	< 0.001	17.80	(4.70 67.35)	< 0.001	16.31	(4.29 61.94)	< 0.001
Male55_59	19.99	(7.54 53.00)	< 0.001	20.28	(7.63 53.87)	< 0.001	27.61	(7.59 100.41)	< 0.001	25.59	(7.04 92.96)	< 0.001
Male60_64	36.14	(13.67 95.57)	< 0.001	36.81	(13.92 97.38)	< 0.001	50.63	(13.77 186.17)	< 0.001	44.62	(12.08 164.77)	< 0.001
Male65_69	54.81	(19.96 150.52)	< 0.001	55.99	(20.34 154.1)	< 0.001	80.85	(21.1 308.8)	< 0.001	77.03	(19.54 303.74)	< 0.001
Male70_74	93.97	(34.90 252.9)	< 0.001	97.80	(36.13 264.7)	< 0.001	138.12	(36.5 521.9)	< 0.001	131.21	(33.64 511.86)	< 0.001
Female < 30	0.43	(0.10 1.85)	0.135	0.42	(0.10 1.84)	0.131	0.56	(0.10 3.25)	0.394	0.37	(0.05 2.71)	0.201
Female30_34	1.55	(0.44 5.51)	0.372	1.54	(0.43 5.47)	0.379	1.87	(0.40 8.81)	0.299	1.87	(0.40 8.80)	0.296
Female35_39	0.89	(0.21 3.74)	0.830	0.89	(0.21 3.76)	0.836	1.29	(0.24 6.96)	0.695	0.85	(0.13 5.56)	0.822
Female40_44	4.29	(1.32 13.98)	0.001	4.34	(1.33 14.17)	0.001	6.10	(1.41 26.40)	0.001	5.20	(1.17 23.03)	0.004
Female45_49	2.85	(0.88 9.16)	0.021	2.89	(0.90 9.32)	0.020	4.02	(0.94 17.26)	0.014	3.43	(0.77 15.37)	0.034
Female50_54	9.31	(3.45 25.15)	< 0.001	9.37	(3.46 25.39)	< 0.001	13.75	(3.65 51.80)	< 0.001	13.50	(3.57 51.09)	< 0.001
Female55_59	14.11	(5.27 37.79)	< 0.001	14.13	(5.27 37.91)	< 0.001	19.26	(5.21 71.17)	< 0.001	18.49	(5.00 68.38)	< 0.001
Female60_64	18.84	(7.14 49.73)	< 0.001	19.00	(7.20 50.14)	< 0.001	25.75	(7.01 94.55)	< 0.001	23.26	(6.32 85.70)	< 0.001
Female65_69	28.75	(10.62 77.79)	< 0.001	29.12	(10.76 78.77)	< 0.001	38.18	(10.2 142.31)	< 0.001	36.36	(9.48 139.44)	< 0.001
Female70_74	47.56	(17.77 127.29)	< 0.001	49.20	(18.39 131.6)	< 0.001	65.80	(17.7 244.67)	< 0.001	61.44	(16.02 235.61)	< 0.001
**Location1**												
PC				ref.			ref.			ref.		
US				0.87	(0.68 1.12)	0.152	0.89	(0.70 1.14)	0.238	0.93	(0.71 1.20)	0.443
AST				0.78	(0.52 1.18)	0.118	0.78	(0.51 1.19)	0.127	0.84	(0.54 1.30)	0.293
RST				0.56	(0.29 1.09)	0.025	0.63	(0.33 1.19)	0.061	0.79	(0.44 1.43)	0.307
VRST				0.60	(0.34 1.05)	0.018	0.60	(0.33 1.09)	0.027	0.65	(0.36 1.17)	0.059
AR				0.83	(0.58 1.20)	0.201	0.92	(0.64 1.34)	0.584	0.97	(0.66 1.42)	0.814
RR				0.51	(0.30 0.87)	0.001	0.59	(0.32 1.06)	0.020	0.58	(0.31 1.07)	0.023
VRR				0.45	(0.25 0.81)	< 0.001	0.54	(0.32 0.92)	0.003	0.53	(0.30 0.94)	0.004
**Social Class**												
Intermediate							ref.			ref.		
Professional							0.37	(0.14 0.93)	0.006	0.27	(0.09 0.82)	0.002
Skilled non-manual							1.07	(0.76 1.51)	0.607	1.08	(0.75 1.55)	0.591
Skilled manual							1.36	(0.97 1.90)	0.020	1.43	(1.00 2.04)	0.009
Partly skilled							1.33	(0.93 1.89)	0.039	1.48	(1.02 2.14)	0.007
Unskilled							1.39	(0.93 2.08)	0.032	1.45	(0.95 2.22)	0.024
**House ownership**												
Owned							ref.			ref.		
Publicly rented							2.10	(1.64 2.70)	< 0.001	2.09	(1.61 2.71)	< 0.001
Privately rented							1.90	(0.99 3.65)	0.011	2.04	(1.05 3.98)	0.006
Others							2.88	(1.27 6.53)	0.001	2.99	(1.23 7.24)	0.001
**Practice char**.												
WTE GPs per 1000 pop.										0.89	(0.44 1.81)	0.670
GPs age (mean)										1.00	(0.97 1.02)	0.911
Survey year 1998										0.93	(0.71 1.22)	0.492
Log pseudo-likelihood	-2338			-2326			-2156			-1945		
	15435											
Sample size				15423			14448			13019		
Joint significance of rural										X^2^(7) = 11.5	p = 0.116	

* *OR *Odds Ratio, *ref*. reference group ^1 ^*PC *Primary Cities, *US *Urban Settlements, *AST *Accessible Small Towns, *RST *Remote Small Towns, *VRST *Very Remote Small Towns, *AR *Accessible Rural, *RR *Remote Rural, *VRR *Very Remote Rural. ^2 ^*p*-values calculated using standard errors clustered by practice

### Hospital admissions due to CHD

In the final model which adjusted for individual and practice characteristics, and year of survey, older age, and public housing tenure was associated with significantly higher levels of admission for CHD than the respective reference group (Table 4 Model 4). There was also some evidence of a relationship between hospital admissions for CHD and living in remote small towns (*p *= 0.009).

**Table 4:** Association between hospital admissions for CHD and location with adjustments for population and practice characteristics

		Model 1			Model 2			Model 3			Model 4	
	OR*	**(99% CI)**	P > z	OR	**(99% CI)**	P > z	OR	**(99% CI)**	P > z	OR	**(99% CI)**	P > z
**Age and sex**												
Male < 30	ref.			ref.			ref.			ref.		
Male30_34	6.21	(0.49 78.15)	0.063	6.25	(0.50 77.82)	0.061	4.75	(0.40 56.48)	0.105	4.75	(0.38 60.17)	0.114
Male35_39	6.12	(0.67 55.92)	0.035	6.15	(0.67 56.24)	0.035	4.90	(0.54 44.21)	0.063	3.01	(0.29 31.08)	0.220
Male40_44	33.55	(4.59 245.36)	< 0.001	33.57	(4.62 243.96)	< 0.001	27.58	(3.69 206.35)	< 0.001	23.22	(3.06 175.95)	< 0.001
Male45_49	74.16	(9.96 551.96)	< 0.001	75.59	(10.16 562.40)	< 0.001	59.18	(8.02 436.71)	< 0.001	53.74	(7.14 404.44)	< 0.001
Male50_54	170.39	(23.19 1251.92)	< 0.001	170.39	(23.45 1238.11)	< 0.001	139.90	(18.76 1043.33)	< 0.001	132.91	(17.38 1016.13)	< 0.001
Male55_59	205.77	(29.19 1450.64)	< 0.001	203.74	(29.14 1424.52)	< 0.001	161.44	(22.47 1160.04)	< 0.001	135.21	(18.49 988.55)	< 0.001
Male60_64	193.14	(27.90 1336.90)	< 0.001	193.89	(28.08 1338.57)	< 0.001	154.88	(21.68 1106.68)	< 0.001	131.83	(18.05 962.63)	< 0.001
Male65_69	161.35	(22.96 1133.66)	< 0.001	174.50	(24.85 1225.56)	< 0.001	139.04	(19.25 1004.24)	< 0.001	169.90	(22.13 1304.20)	< 0.001
Male70_74	163.06	(22.48 1182.70)	< 0.001	184.75	(25.31 1348.78)	< 0.001	156.26	(20.71 1178.90)	< 0.001	181.49	(22.35 1473.79)	< 0.001
Female < 30	0.00	(0.00 0.00)	< 0.001	0.00	(0.00 0.00)	< 0.001	0.00	(0.00 0.00)	< 0.001	0.00	(0.00 0.00)	< 0.001
Female30_34	1.48	(0.11 20.48)	0.699	1.48	(0.11 20.52)	0.699	1.23	(0.09 17.22)	0.843	1.16	(0.08 16.39)	0.883
Female35_39	9.63	(1.10 84.70)	0.007	9.76	(1.11 85.62)	0.007	8.34	(0.94 73.95)	0.012	5.29	(0.51 54.44)	0.065
Female40_44	7.03	(0.82 60.24)	0.019	7.21	(0.85 61.37)	0.017	6.18	(0.74 51.59)	0.027	5.94	(0.70 50.15)	0.031
Female45_49	16.62	(2.18 126.56)	< 0.001	17.00	(2.24 129.15)	< 0.001	11.72	(1.54 89.09)	0.002	11.38	(1.46 88.97)	0.002
Female50_54	35.63	(4.85 262.00)	< 0.001	35.93	(4.89 264.15)	< 0.001	30.84	(4.15 228.96)	< 0.001	28.37	(3.81 211.34)	< 0.001
Female55_59	73.93	(10.25 533.24)	< 0.001	75.52	(10.53 541.46)	< 0.001	51.40	(7.27 363.54)	< 0.001	41.82	(5.76 303.45)	< 0.001
Female60_64	80.94	(11.24 583.04)	< 0.001	80.31	(11.20 575.85)	< 0.001	65.97	(9.06 480.42)	< 0.001	57.36	(7.57 434.48)	< 0.001
Female65_69	60.50	(8.11 451.47)	< 0.001	67.63	(9.01 507.71)	< 0.001	48.66	(6.41 369.24)	< 0.001	53.11	(6.58 428.91)	< 0.001
Female70_74	77.00	(10.30 575.79)	< 0.001	81.05	(10.83 606.71)	< 0.001	69.78	(9.06 537.59)	< 0.001	89.42	(10.84 737.58)	< 0.001
**Location1**												
PC				ref.			ref.			ref.		
US				0.94	(0.65 1.36)	0.649	1.03	(0.71 1.48)	0.860	1.08	(0.74 1.57)	0.666
AST				0.89	(0.52 1.53)	0.572	0.98	(0.55 1.73)	0.916	0.99	(0.57 1.71)	0.878
RST				0.37	(0.16 0.86)	0.002	0.41	(0.16 1.03)	0.013	0.34	(0.10 1.14)	0.009
VRST				0.65	(0.13 3.16)	0.486	0.70	(0.10 5.04)	0.646	1.06	(0.12 9.60)	0.995
AR				0.69	(0.40 1.18)	0.077	0.78	(0.45 1.35)	0.246	0.83	(0.48 1.42)	0.326
RR				0.68	(0.35 1.35)	0.152	0.86	(0.41 1.84)	0.621	0.96	(0.45 2.08)	0.735
VRR				0.49	(0.26 0.90)	0.003	0.57	(0.31 1.03)	0.015	0.70	(0.37 1.35)	0.087
**Social Class**												
Intermediate							ref.			ref.		
Professional							0.61	(0.27 1.36)	0.112	0.58	(0.26 1.28)	0.073
Skilled non-manual							0.99	(0.61 1.61)	0.959	0.99	(0.60 1.64)	0.935
Skilled manual							1.27	(0.81 1.97)	0.171	1.17	(0.74 1.85)	0.358
Partly skilled							1.19	(0.75 1.91)	0.331	1.16	(0.71 1.91)	0.450
Unskilled							1.36	(0.75 2.46)	0.182	1.28	(0.67 2.45)	0.315
**House ownership**												
Owned							ref.			ref.		
Publicly rented							1.75	(1.23 2.51)	< 0.001	1.75	(1.19 2.57)	< 0.001
Privately rented							1.14	(0.41 3.22)	0.738	1.24	(0.41 3.74)	0.605
Others							1.81	(0.66 5.00)	0.131	2.02	(0.71 5.80)	0.095
**Practice char**.												
WTE GPs per 1000 pop.										1.32	(0.59 2.96)	0.382
GPs age (mean)										0.99	(0.95 1.02)	0.271
Survey year 1998										0.57	(0.39 0.83)	0.110
Log pseudo-likelihood	-2806			-2799			-2710			-2425		
Sample size	15435			15423			14448			13019		
Joint significance of rural										X^2^(7) = 9.84	p = 0.197	

* *OR *Odds Ratio *ref*. reference group ^1 ^*PC *Primary Cities, *US *Urban Settlements, *AST *Accessible Small Towns, *RST *Remote Small Towns, *VRST *Very Remote Small Towns, *AR *Accessible Rural, *RR *Remote Rural, *VRR *Very Remote Rural. ^2 ^*p*-values calculated using standard errors clustered by practice

### Total hospital stay

As expected, older individuals had more hospital stays than younger individuals, in both sexes (Table 5 Model 4). Individuals living in public housing had significantly more hospital stays than people who owned their house. Adjusted for individual and practice characteristics, there was no association between total hospital stays and place of residence.

**Table 5:** Association between total hospital stays and location with adjustments for population and practice characteristics

		Model 1			Model 2			Model 3			Model 4	
	OR*	**(99% CI)**	P > z	OR	**(99% CI)**	P > z	OR	**(99% CI)**	P > z	OR	**(99% CI)**	P > z
**Age and sex**												
Male < 30	ref.			ref.			ref.			ref.		
Male30_34	1.89	(0.70 5.09)	0.099	1.84	(0.74 4.56)	0.084	1.52	(0.77 2.99)	0.115	1.44	(0.71 2.93)	0.204
Male35_39	1.22	(0.80 1.87)	0.228	1.21	(0.80 1.84)	0.230	1.23	(0.78 1.95)	0.247	1.08	(0.58 2.01)	0.753
Male40_44	2.75	(1.00 7.51)	0.010	2.69	(1.08 6.75)	0.005	1.79	(1.03 3.13)	0.007	1.85	(0.99 3.48)	0.013
Male45_49	2.12	(1.22 3.67)	< 0.001	2.12	(1.23 3.64)	< 0.001	1.90	(1.12 3.24)	0.002	1.80	(0.99 3.25)	0.011
Male50_54	2.91	(1.76 4.79)	< 0.001	2.87	(1.79 4.60)	< 0.001	2.68	(1.65 4.36)	< 0.001	2.65	(1.45 4.90)	< 0.001
Male55_59	3.93	(2.68 5.78)	< 0.001	3.97	(2.73 5.80)	< 0.001	3.76	(2.46 5.74)	< 0.001	4.08	(2.35 6.90)	< 0.001
Male60_64	6.49	(3.94 10.68)	< 0.001	6.57	(3.97 10.88)	< 0.001	6.35	(3.60 11.20)	< 0.001	5.82	(3.21 10.68)	< 0.001
Male65_69	5.29	(3.53 7.91)	< 0.001	5.38	(3.65 7.94)	< 0.001	4.96	(3.25 7.59)	< 0.001	9.02	(5.22 15.58)	< 0.001
Male70_74	7.57	(5.01 11.44)	< 0.001	7.82	(5.23 11.70)	< 0.001	6.90	(4.48 10.63)	< 0.001	12.11	(6.91 21.06)	< 0.001
Female < 30	0.96	(0.62 1.47)	0.787	0.98	(0.64 1.49)	0.887	0.82	(0.51 1.33)	0.291	0.72	(0.41 1.26)	0.130
Female30_34	1.90	(1.24 2.90)	< 0.001	1.82	(1.20 2.75)	< 0.001	1.37	(0.83 2.25)	0.104	1.18	(0.64 2.12)	0.500
Female35_39	1.71	(1.10 2.67)	0.002	1.69	(1.11 2.56)	0.001	1.39	(0.89 2.16)	0.056	1.50	(0.80 2.75)	0.098
Female40_44	2.01	(1.26 3.19)	< 0.001	2.01	(1.29 3.14)	< 0.001	1.82	(1.12 2.96)	0.002	1.63	(0.94 2.80)	0.022
Female45_49	2.58	(1.57 4.26)	< 0.001	2.65	(1.61 4.37)	< 0.001	2.51	(1.41 4.46)	< 0.001	1.96	(1.06 3.64)	0.005
Female50_54	3.03	(1.99 4.62)	< 0.001	3.05	(2.03 4.57)	< 0.001	2.92	(1.85 4.63)	< 0.001	3.35	(1.83 5.94)	< 0.001
Female55_59	4.04	(2.78 5.89)	< 0.001	4.02	(2.80 5.78)	< 0.001	3.17	(2.12 4.75)	< 0.001	3.50	(2.01 6.11)	< 0.001
Female60_64	4.50	(2.82 7.19)	< 0.001	4.50	(2.90 6.98)	< 0.001	3.81	(2.48 5.83)	< 0.001	3.87	(2.28 6.52)	< 0.001
Female65_69	4.70	(2.88 7.68)	< 0.001	4.72	(2.92 7.63)	< 0.001	4.40	(2.43 7.96)	< 0.001	7.70	(3.67 16.15)	< 0.001
Female70_74	8.51	(5.73 12.64)	< 0.001	8.53	(5.81 12.51)	< 0.001	8.04	(5.12 12.63)	< 0.001	14.02	(7.78 25.24)	< 0.001
**Location1**												
PC				ref.			ref.					
US				0.85	(0.69 1.06)	0.061	0.87	(0.69 1.08)	0.098	0.86	(0.69 1.08)	0.088
AST				0.99	(0.65 1.49)	0.934	0.85	(0.60 1.19)	0.204	0.97	(0.65 1.46)	0.841
RST				0.71	(0.43 1.19)	0.089	0.69	(0.44 1.09)	0.037	0.89	(0.50 1.51)	0.496
VRST				1.25	(0.72 2.17)	0.301	1.08	(0.71 1.67)	0.625	1.26	(0.67 2.32)	0.364
AR				0.67	(0.46 0.98)	0.006	0.69	(0.51 0.95)	0.003	0.80	(0.57 1.08)	0.054
RR				0.80	(0.46 1.37)	0.286	1.08	(0.55 2.12)	0.777	1.39	(0.54 3.49)	0.388
VRR				0.93	(0.60 1.44)	0.659	1.04	(0.73 1.48)	0.791	0.97	(0.54 1.38)	0.433
**Social Class**												
Intermediate							ref.			ref.		
Professional							0.42	(0.27 0.63)	< 0.001	0.39	(0.26 0.58)	< 0.001
Skilled non-manual							0.93	(0.69 1.25)	0.534	1.00	(0.73 1.36)	0.988
Skilled manual							0.95	(0.72 1.28)	0.682	1.00	(0.74 1.36)	0.989
Partly skilled							1.07	(0.77 1.49)	0.637	1.24	(0.85 1.81)	0.139
Unskilled							1.24	(0.84 1.86)	0.154	1.28	(0.85 1.93)	0.130
**House ownership**												
Owned							ref.			ref.		
Publicly rented							2.19	(1.70 2.74)	< 0.001	2.18	(1.69 2.81)	< 0.001
Privately rented							1.12	(0.70 1.71)	0.519	1.16	(0.71 1.90)	0.443
Others							1.44	(0.81 2.19)	0.058	1.36	(0.74 2.58)	0.185
**Practice char**.												
WTE GPs per 1000 pop.										1.31	(0.64 2.68)	0.332
GPs age (mean)										1.01	(0.99 1.04)	0.164
Survey year 1998										0.37	(0.30 0.45)	0.201
Log pseudo-likelihood	-36195			-36152			-33270			-29764		
Sample size	15435			15423			14448			13019		
Joint significance of rural										X^2^(7) = 8.64	p = 0.278	

* *OR *Odds Ratio *ref*. reference group ^1 ^*PC *Primary Cities, *US *Urban Settlements, *AST *Accessible Small Towns, *RST *Remote Small Towns, *VRST *Very Remote Small Towns, *AR *Accessible Rural, *RR *Remote Rural, *VRR *Very Remote Rural. ^2 ^*p*-values calculated using standard errors clustered by practice

## Discussion

Our analyses of four health outcomes using individual-based data adjusted for the characteristics of people living in different parts of Scotland and the general practices serving them, failed to reveal a consistent pattern of substantially different health among those living in rural areas compared with primary cities. Older age and living in publicly owned housing appeared to be more important determinants of health than rurality or structure of the practices serving the population.

Producing information about the socio-demographic characteristics of individuals living in different urban-rural areas, the practices serving them and relevant health outcomes is currently not straightforward because of the lack of a dataset that contains everything. We have shown that it is possible to link different routinely collected datasets to explore urban-rural issues. An alternative approach would be to conduct specific large-scale epidemiological surveys, which tend to be more expensive.

The health surveys used in our study were nationally representative, so should produce more representative results for Scotland as a whole than studies representing particular groups or places. The SHS linked to the SEURC over-sampled rural areas in order to provide sufficient sample sizes within each region. The SHS also provided Sampling weights to account for the sampling design and non-response bias. These gave us enough population in remote rural areas and made our results robust. We overcame the limitation of area-based analyses by looking at individual-based socio-economic characteristics.

To the best of our knowledge, this is the first study to examine the association between rurality and health in Scotland, after adjusting for the demographic and socio-economic characteristics of the individuals living in different areas, and the characteristics of the general practices serving them. The few international studies that adjusted for the structure of care have tended to use area-based aggregated data rather than individual-based information [52,64-67]. This is in part due to lack of relevant data.

A limitation of the study was the small number of health indicators available for analysis. Health is a complex issue, with many factors influencing health status. Rurality is one factor that has been proposed as an important influence on health, but it is not easy to ascertain whether place or other physical, social and cultural environmental factors (e.g., pollution, traffic and neighborhood noise) are important. We were unable to examine any of these factors. Furthermore, in addition to demographic and socio-economic factors, there are other important influences on health, such as drinking alcohol, smoking and substance abuse. We were unable to determine whether these potential confounding variables affected our results. As wellbeing is a combination of physical and mental health, adjusting for psychological problems might have produced different results.

Routine data are primarily collected for administrative purposes and their accuracy has been questioned [68]. Population-based, administrative data sets have been used to assess service utilization - among other outcomes - for many years, and the data linkage and analysis procedures have been validated and well-established [69,70]. The use of administrative data eliminates biases associated with the use of self-report data and attrition problems common in studies involving long-term follow-up. On the other hand, routinely collected administrative data can suffer from problems with completeness and accuracy data collection, issues which are not under the direct control of researchers and which can be difficult to quantify [71].

Most previous studies of rural-urban differences in health have adjusted for the demographic and/or socio-economic characteristics of the population [12,72]. Few studies in the US, and only one study in the UK, have allowed for the structure of health care providers [52,64-67]. This is in part due to lack of relevant data. Studies in the US have found higher mortality in areas with fewer primary care doctors [64-67]. The study in England concluded that mortality levels were weakly associated with the characteristics of practices delivering primary medical care [52]. That study used health authority aggregated data about the structure of care. Such aggregation might mask the characteristics of individual general practitioners or practices and thereby affect overall results. In our study, the practice characteristics assessed were not associated with the health outcomes measured. Using individual-based data, we did not find strong or consistent significant associations between the various health indicators assessed and location, after allowing for population and practice characteristics.

## Conclusions

Compositional determinants of health (age and gender) and socio-economic characteristics were found to be more strongly associated with the health outcomes examined than contextual factors (including rurality). Similar studies, using more health measures, should be carried out to confirm or refute our findings.

## Competing interests

The authors declare that they have no competing interests.

## Authors' contributions

**PT **This study was conducted as part of a PhD thesis by PT. PT developed the idea for the study and conducted all aspects of the study under the supervision of PH and MS. PT's PhD was funded by the Chief Scientist Office of the Scottish Executive Department of Health. **PH **is funded by NHS Grampian. **MS **was funded by the Chief Scientist Office. All authors were involved in the final review of the manuscript and all authors read and approved the final manuscript.

## Pre-publication history

The pre-publication history for this paper can be accessed here:

http://www.biomedcentral.com/1472-6963/12/43/prepub
